# Few-shot learning to identify atypical endometrial hyperplasia and endometrial cancer based on transvaginal ultrasonic images

**DOI:** 10.1016/j.heliyon.2024.e36426

**Published:** 2024-08-16

**Authors:** Mingyue Wang, Wen Liu, Xinxian Gu, Feng Cui, Jin Ding, Yindi Zhu, Jinyan Bian, Wen Liu, Youguo Chen, Jinhua Zhou

**Affiliations:** a Department of Obstetrics and Gynecology, The First Affiliated Hospital of Soochow University, Suzhou, China; b Department of Gastroenterology, Changzhou Hospital of Traditional Chinese Medicine, China; c Department of Ultrasound, The Fourth Affiliated Hospital of Soochow University, Suzhou, China; d Jiangsu Province Engineering Research Center of Precision Diagnostics and Therapeutics Development, Soochow University, Suzhou, China; e Department of Ultrasound, The Hospital of Traditional Chinese Medicine, Suzhou, China

**Keywords:** Atypical endometrial hyperplasia, Endometrial cancer, Ultrasound images, Machine learning, Deep learning, Few-shot learning

## Abstract

**Objective:**

It is challenging to accurately distinguish atypical endometrial hyperplasia (AEH) and endometrial cancer (EC) under routine transvaginal ultrasonic (TVU) detection. Our research aims to use the few-shot learning (FSL) method to identify non-atypical endometrial hyperplasia (NAEH), AEH, and EC based on limited TVU images.

**Methods:**

The TVU images of pathologically confirmed NAEH, AEH, and EC patients (n = 33 per class) were split into the support set (SS, n = 3 per class) and the query set (QS, n = 30 per class). Next, we used dual pretrained ResNet50 V2 which pretrained on ImageNet first and then on extra collected TVU images to extract 1*64 eigenvectors from the TVU images in SS and QS. Then, the Euclidean distances were calculated between each TVU image in QS and nine TVU images of SS. Finally, the k-nearest neighbor (KNN) algorithm was used to diagnose the TVU images in QS.

**Results:**

The overall accuracy and macro precision of the proposed FSL model in QS were 0.878 and 0.882 respectively, superior to the automated machine learning models, traditional ResNet50 V2 model, junior sonographer, and senior sonographer. When identifying EC, the proposed FSL model achieved the highest precision of 0.964, the highest recall of 0.900, and the highest F1-score of 0.931.

**Conclusions:**

The proposed FSL model combining dual pretrained ResNet50 V2 eigenvectors extractor and KNN classifier presented well in identifying NAEH, AEH, and EC patients with limited TVU images, showing potential in the application of computer-aided disease diagnosis.

## 1 Introduction

Endometrial cancer (EC), as one of the most common malignancies in the female reproductive tract, accounts for approximately 5 % of female cancer cases globally, seriously threatening the life and health of women [[1], [2], [3]]. It is estimated to have an incidence of 21.4 per 100,000 in North America and 16.6 per 100,000 in Europe, with over 300,000 new cases diagnosed annually worldwide [[4], [5], [6]]. Referring to the 2014 revision of the WHO Classification of Tumors of Female Reproductive Organs, endometrial hyperplasia (EH) is classified into non-atypical endometrial hyperplasia (NAEH) and atypical endometrial hyperplasia (AEH) based on the presence of cytologic atypia [7]. NAEH can be treated conservatively as a benign lesion, with less than 5 % of the cases progressing into EC [8]. Atypical endometrial hyperplasia (AEH), as a precursor lesion of EC, is defined as glands that exhibit various degrees of nuclear atypia and loss of polarity. Previous research revealed that 20 %–50 % of AEH cases may progress to or co-exist with endometrioid EC without treatment [[9], [10], [11]]. Treatment planning, surveillance, and prognosis are significantly different in these three diseases (NAEH, AEH, and EC), so early diagnosis is crucial. Transvaginal ultrasound (TVU) is currently the first-line intrauterine detection method for the early diagnosis of endometrial diseases. TVU presents strengths in well-tolerated, non-invasive, and low-cost compared to diagnostic curettage or hysteroscopic curettage [12,13]. However, this technique is limited by the examiner's experience and the evaluation results may have considerable variability when observing the same ultrasound images between different sonographers. Previous research revealed that the accuracy varies between 82 % and 92 % for the less and more experienced observers when applying transvaginal ultrasound to differentiate adnexal masses [14]. Therefore, developing an objective and highly-accurate diagnostic model is necessary.

Recently, artificial intelligence (AI) achieved rapid development and its application in the medical field is currently a hot topic. Radiomics can transform medical images into diggable data by the high-throughput extraction of quantitative features [15]. Multiply research made use of the extracted radiomics features from ultrasound images, computed tomography (CT), MRI images, PET/CT images, etc for cancer diagnosis and prognosis [[16], [17], [18]]. However, radiomics features are handcrafted and restricted to the operator's expertise. Deep learning (DL), as one of the most powerful and widely applied AI algorithms, can automatically extract image features via multiple processing layers [19]. DL removes the step of regions of interest (ROI) drawing and can provide more in-depth features but requires large datasets. In contrast, the few-shot learning (FSL), as its name indicates, learns from a small base of labeled samples and makes accurate judgments for novel unseen samples. Just as the enhancement of our human being learning ability, FSL can acquire improved identification ability from other larger datasets and then be applied to new tasks which is called meta-learning [20].

This study involved building automated machine learning (AutoML) models, traditional DL models, and FSL models to classify NAEH, AEH, and EC. Furthermore, we invited two sonographers to conduct the ternary classification task and compared their performance with these computer-aided disease classification tools.

## 2 Methods

### 2.1 Datasets

Three datasets were used in this study: Dataset 1 was the TVU images of NAEH, AEH, and EC collected from the First Affiliated Hospital of Soochow University (n = 100 per class). Dataset 2 was the TVU images of NAEH, AEH, and EC collected from the Suzhou Hospital of Traditional Chinese Medicine (n = 33 per class). Dataset 3 was the TVU images of normal uterus and uterus myoma collected from the First Affiliated Hospital of Soochow University (n = 300 per class).

**Fig. 1:**
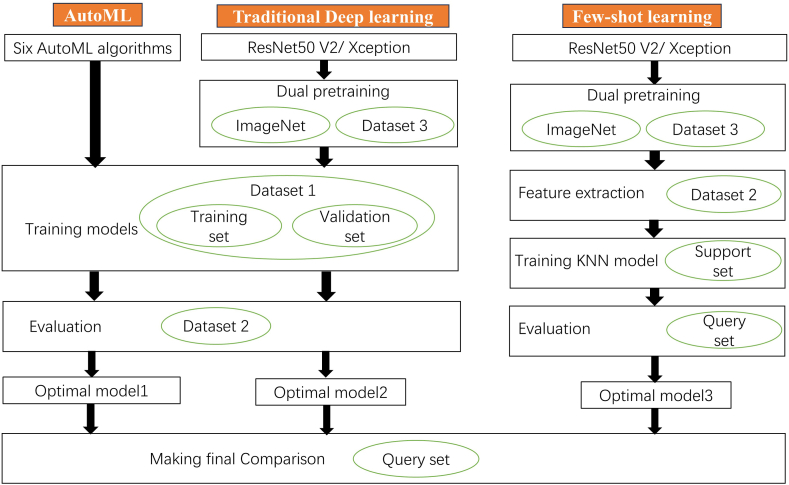
The flow chart of the study. AutoML, automated machine learning; DL, deep learning; FSL, few-shot learning.

Dataset 2 was split into the support set (SS, n = 3 per class) and query set (QS, n = 30 per class) for training and evaluation of the FSL models. We explained the SS and QS below. The QS was also used as a final test set to compare all models. The concrete applied process of datasets can be seen in Fig. 1. The institutional review board of the First Affiliated Hospital of Soochow University has approved this retrospective research.

### 2.2 Imaging acquisition and ROI segmentation

TVU images were acquired from different ultrasound machines in two hospitals, including the TOSHIBA Aplio400, SIEMENS Acuson juniper, GE VolusonE6, GE VolusonE8, and GE VolusonE10. All TVU images were stored in DICOM format and then converted to PNG format. We selected the TVU images with the most noticeable lesions to represent each patient. During the process of TVU image collection, we applied the following exclusion criteria: (1) the TVU images revealed multiple uterine disorders; (2) the TVU images were blurred or had obvious defects.

All TVU images were preprocessed and the abnormal region was masked as the ROI using the image processing toolbox on MatLab (version: R2021b; Natick, MA). Three sonographers who were blinded to the clinical-pathological data and had at least 5 years of experience participated in confirming the ROI. One sonographer was responsible for manually delineating the ROI and the other two radiologists acted as reviewers and made corrections by consensus.

### 2.3 The handcrafted radiomics feature extraction and AutoML modeling

**Fig. 2:**
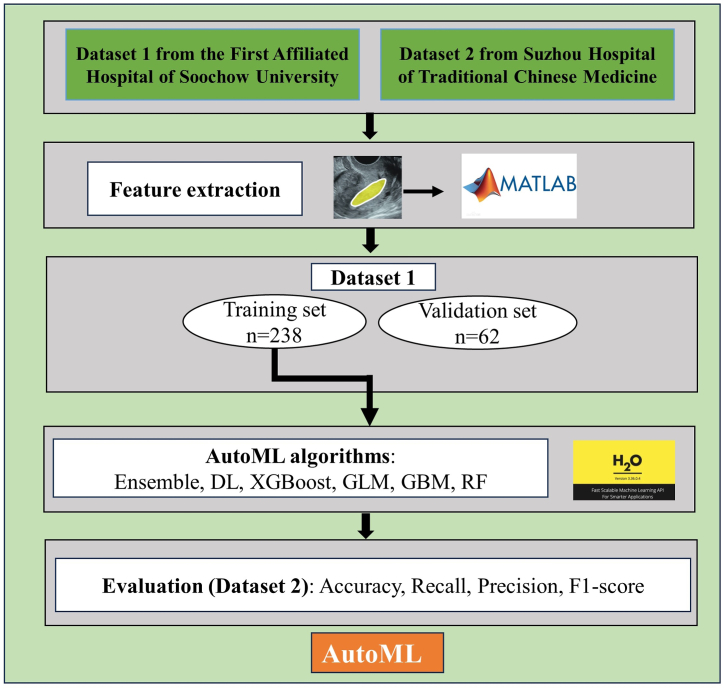
The AutoML modelling and evaluation process. AutoML, automated machine learning.

A total of 33 handcrafted radiomics features were extracted from each ROI of the TVU images in Datasets 1 and 2 using MatLab (version: R2021b; Natick, MA), including texture features based on the grey histogram (GH) (n = 6), texture features based on grey level co-occurrence matrix (GLCM) (n = 6), Gabor filter features (GB) (n = 3), Gauss Markov Random Field features (GMRF) (n = 12) and Tamura features (T) (n = 6). Then, Dataset 1 was split into the training set (n = 238) and validation set (n = 62). The training set was applied to develop models based on six AutoML algorithms including Ensemble, Deep learning (DL), eXtreme Gradient Boosting (XGBoost), generalized linear model (GLM), Gradient Boosting Machine (GBM), and random forest (RF) from the H2O platform (www.h2o.ai). H2O, as an open-source and extensible platform for machine learning (ML), can provide various ML algorithms, automatically adjust hyperparameters, and use K-fold cross-validation to validate models [21]. Five-fold cross-validation was performed to obtain models with the minimum mean square error (MSE). Finally, the developed AutoML models were evaluated in Dataset 2 to identify the best-performing model (Fig. 2).

### 2.4 The traditional DL models

The DL model was constructed based on the architecture of ResNet50 V2. Residual Network is a well-known and widely-used CNN architecture for image classification [22]. As an improved version of the original ResNet50, ResNet50 V2 captures more fine details and acquires higher accuracy [23,24]. A dual pretrained ResNet50 V2 model was trained to identify the NAEH, AEH, and EC. First, the base ResNet50 V2 was pretrained on ImageNet (https://image-net.org/). Then, the architecture and learned weights of the convolutional layers were retained for the second pretraining in Dataset 3, which is typically called transfer learning. The fully connected layer (FCL) and the classified layer (1000 categories of ImageNet) of the first pretrained ResNet50 V2 were truncated and replaced with four new full-connected layers (1*1024, 1*512, 1*128, 1*64), and a new classifier layer of two categories (the normal uterus and uterus myoma in Dataset 3). Subsequently, the architecture and learned weights of the dual pretrained ResNet50 V2 were retained and the classified layer was replaced with a new classifier layer of three categories (NAEH, AEH, and EC). Finally, Dataset 1 was split into the training set (n = 240) and validation set (n = 60) to train the dual pretrained ResNet50 V2 model to identify NAEH, AEH, and EC. Dataset 2 was applied to evaluate the performance of the established dual pretrained ResNet50 V2 model.

Before the model pretraining and classification, the selected TVU images were normalized to the range (−1, 1) using a min-max transformation and resized to 331 * 331 pixels. In addition, to avoid overfitting and improve the generalization of the traditional DL model, data augmentation was applied in the training set, such as crop, rotation, flip, and color. The hyperparameters were as follows: a batch size of 32, the epoch was equal to 30 with an early stop policy, an initial learning rate of 0.001, the first seven epochs were the initial learning rate, multiplying to 0.1 every 7 epochs and the Optimizer was Adam. Cross-entropy was used as the loss function.

**Fig. 3:**
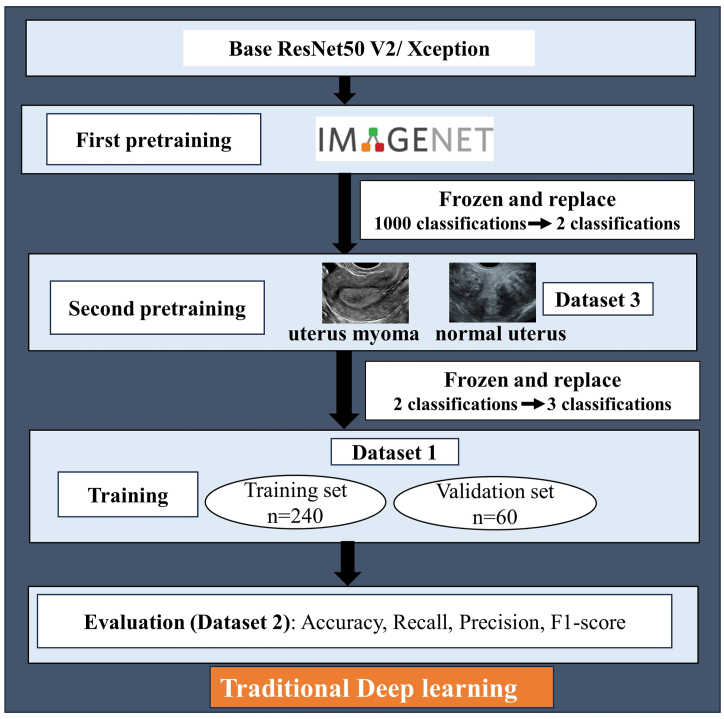
The DL model development and evaluation process. DL, deep learning.

Furthermore, we used the Xception architecture to replace the ResNet50 V2 and restart the above pretraining and training steps. The process of DL model development and evaluation shown in Fig. 3.

### 2.5 Few-shot learning

FSL was applied to accurately identify samples from target classes with only a few annotated training samples per class [25]. The annotated training samples in target classes refer to the Support set (SS) and the unlabeled samples in target classes refer to the query set (QS). The SS consisted of N base classes and each class included K samples, namely, N-way K-shot classification. In this research, the SS consisted of three classes, and each class contained three images that were randomly selected from Dataset 2, namely, 3-way 3-shot classification. The remaining images in Dataset 2 were treated as the QS.

The dual pretraining process as the traditional DL model, we retained the architecture and learned weights of the dual pretrained ResNet50 V2 and Xception respectively, and used as a feature extractor for TVU images in SS and QS. The 1*64 eigenvector was outputted to represent each TVU image in SS and QS. The detailed process of dual pretraining and feature extraction is provided in Supplementary Materials Fig. S1. Then, the Euclidean distances were calculated between each TVU image in the QS and the nine SS images. The acquired nine Euclidean distance values were input into the k-nearest neighbor (KNN) classifier to decide the final classification for each TVU image in QS.

**Fig. 4:**
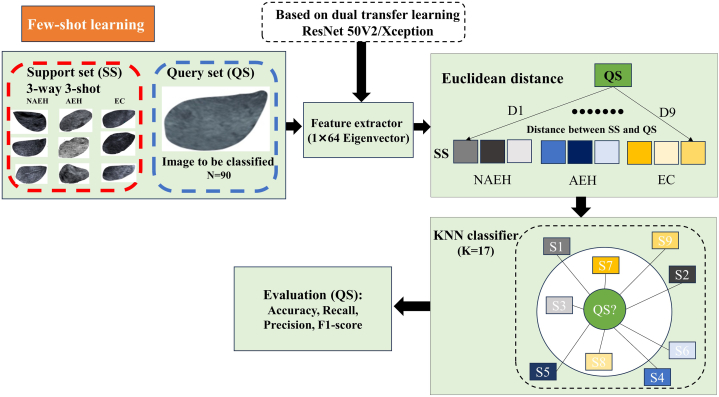
The FSL model establishment and evaluation process. FSL, few-shot learning; KNN, k-nearest neighbor classifier; SS, support set; QS, query set.

Euclidean distance, as one of the most common distance metrics, reflects the absolute distance between two points in a multi-dimensional space as follows:

$$D\left(s,q\right)=\sqrt{{\left(s_{1}-q_{1}\right)}^{2}+{\left(s_{2}-q_{2}\right)}^{2}+\cdots +{\left(s_{n}-q_{n}\right)}^{2}}=\sqrt{\sum_{i=1}^{n}{\left(s_{i}-q_{i}\right)}^{2}}$$

where D represents the Euclidean distance, s represents one of the labeled images in SS, q represents one of the unlabeled images in QS, and n represents the dimensionality of the extracted feature vectors. The KNN algorithm, as one of the oldest and simplest methods for pattern classification, is based on the rule that classifies each unlabeled example by the majority label among its k-nearest neighbors in the training set [26]. Nine labeled TVU images in SS were treated as the training set and the category of each TVU image in QS was determined by finding the majority label among its k-nearest neighbors in SS using the Euclidean distance. The FSL model establishment and evaluation are shown in Fig. 4.

### 2.6 Model interpretation

The AutoML model was visualized by the LIME algorithm (Local interpretable model-agnostic explanations) which can interpret the predictions of any classifier or regressor by making local approximations using an explainable model [27]. It modifies individual data samples by adjusting the eigenvalues and observing the impact on the output, acting as an "interpreter" to explain the predictions. The LIME output is a set of interpretations that represent the contribution of each feature to the prediction of a single sample, a kind of local interpretability.

The traditional DL model and FSL model were visualized by the Gradient-weighted Class Activation Mapping (Grad-CAM) algorithm [28]. It generates TVU image location heatmaps produced by the gradients of the convolutional layer undergoing dual pretraining. The closer the color of the lesion in the heatmap to red, the greater the weight, whereas the closer the color to blue, the smaller the weight.

### 2.7 Evaluation metrics

The model's performance was evaluated by the accuracy, recall, precision, and F1-score according to the following equations: $Accuracy=\frac{\text{TP}+\text{TN}}{\text{TP}+\text{TN}+\text{FN}+\text{FP}}$; $\text{Recall}=\frac{\text{TP}}{\text{TP}+\text{FN}}$; $\text{Precision}=\frac{\text{TP}}{\text{TP}+\text{FP}}$; $F1-\text{score}=\frac{2\times \text{Precision}\times \text{Recall}}{\text{Precision}+\text{Recall}}$. TN, FN, TP, and FP represent true negatives, false negatives, true positives, and false positives, respectively.

Given that this study was to conduct a ternary classification task based on balanced datasets, the macro precision, macro recall, and macro F1-score were also obtained by calculating the averages of the same metrics, used to evaluate the overall performance of models. The value of accuracy was equal to the macro recall due to the balanced datasets.

### 2.8 Comparing the performance of the AutoML models, traditional DL models, FSL models, and sonographers

The QS was treated as a final test set to compare the performance of the optimal AutoML models, traditional DL models, and FSL models. Furthermore, a senior sonographer with over fifteen years of experience and a junior sonographer with less than five years of experience were invited to imitate the learning steps of the FSL framework to demonstrate the benefits of practical clinical application of the computer-aided tools. Nine TVU images from SS were provided to the two sonographers as a reference and the remaining 90 images from QS were used to classify and evaluate.

The H2O package in R software installed from the H2O.ai platform (www.h2o.ai) was used to establish the AutoML models. Python software (version 3.8.8) and TensorFlow (version: 2.8.0) were used to fit the traditional DL and FSL models. Statistical analysis was performed using R software (version 4.1.0).

## 3 Results

### 3.1 Baseline characteristics of dataset 1 and dataset 2

Dataset 1 contained 100 NAEH patients, 100 AEH patients, and 100 EC patients with an average age of 45.4, 48.2, and 58.1 years respectively which were significantly different (P ＜0.001). In addition, the number of menopausal patients was significantly different (19, 35, and 61 respectively; P ＜0.001). Dataset 2 comprised 33 NAEH patients, 33 AEH patients, and 33 EC patients with an average age of 46.7, 50.1, and 57.5 years respectively which were significantly different (P = 0.002). The number of menopausal patients was 6, 12, and 21 respectively, which was also significantly different (P ＜0.001). The other baseline characteristics are summarized in Table 1.

**Table 1:** Baseline characteristics of patients in Dataset1 and Dataset2.

Variables	Dataset1			P	Dataset2			P
	NAEH (n = 100)	AEH (n = 100)	EC (n = 100)		NAEH (n = 33)	AEH (n = 33)	EC (n = 33)	
Age (mean (SD))	45.4 ± 7.5	48.2 ± 10.2	58.1 ± 11.0	<0.001	46.7 ± 6.9	50.1 ± 12.3	57.5 ± 10.7	0.002
Menopause (n)	19	35	61	<0.001	6	12	21	<0.001
BMI (mean (SD))	24.5 ± 4.5	25.1 ± 5.2	25.3 ± 5.1	0.493	24.1 ± 4.6	25.3 ± 5.5	25.7 ± 5.0	0.411
Parity (mean (SD))	1.23 ± 0.31	1.2 ± 0.29	1.24 ± 0.33	0.683	1.34 ± 0.34	1.29 ± 0.33	1.39 ± 0.40	0.528
Hypertension (n)	15	26	28	0.063	5	9	11	0.226
T2DM (n)	8	11	17	0.137	2	5	6	0.316

BMI, Body Mass Index; T2DM, Diabetes mellitus type 2.

### 3.2 Performance of the six AutoML models

Supplementary Materials Table S1 summarized the overall performance of six AutoML models in the ternary classification task in Dataset 2. Supplementary Materials Table S2 summarized the precision, recall, and F1-score of six AutoML models in identifying each of these three categories (NAEH, AEH, EC) in Dataset 2. The results indicate that the Ensemble model achieved the best performance with an accuracy of 0.758, a macro precision of 0.771, and a macro F1-score of 0.759. When identifying AEH, the XGBoost model achieved the highest precision of 0.735 and the highest F1-score of 0.746, with the Ensemble and DL models achieved the highest recall of 0.788. When identifying EC, the Ensemble model achieved the highest precision, recall, and F1-score among the six algorithms (0.885, 0.697, and 0.780 respectively).

Four NAEH patients, four AEH patients, and four EC patients were randomly selected from Dataset 2 to make visual interpretations for the optimal Ensemble model using the LIME algorithm (Supplementary Materials Figure S2 A-C). The LIME graphs of the Ensemble model revealed how the important radiomics features extracted from the TVU images contributed to the diagnosis of NAEH, AEH, and EC. The red color represents the negative impact on the outcome and the blue color represents the positive impact on the outcome.

### 3.3 Performance of the traditional DL models

Supplementary Materials Table S3 and Table S4 summarized the performance of the two DL models in Dataset 2. The dual pretrained ResNet50 V2 model and Xception model achieved the same accuracy of 0.737 in the ternary classification task. However, the dual pretrained ResNet50 V2 model had a higher macro precision and macro F1-score compared with the dual pretrained Xception model (macro precision 0.757vs0.741, macro F1-score 0.738vs0.737). When identifying AEH, the dual pretrained ResNet50 V2 achieved a precision of 0.636, a recall of 0.848, and an F1-score of 0.727. When identifying EC, the dual pretrained ResNet50 V2 achieved a precision of 0.778, a recall of 0.636, and an F1-score of 0.700.

**Fig. 5:**
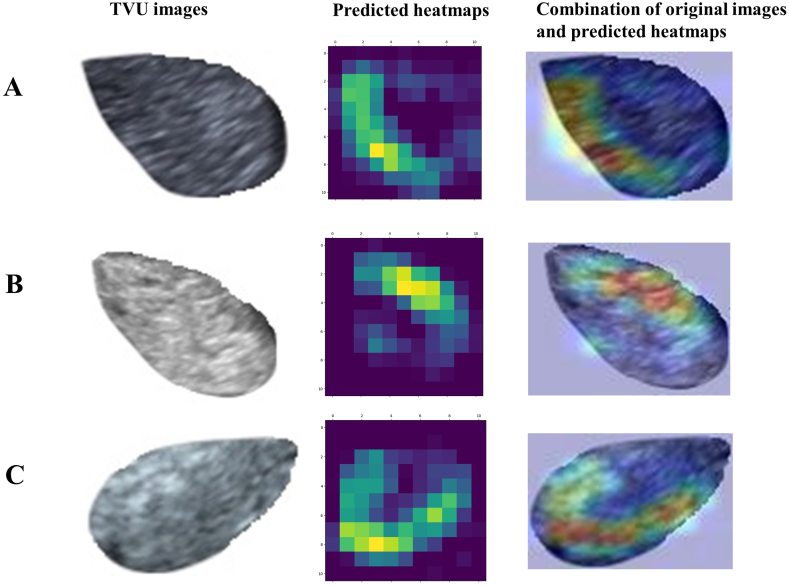
Heatmaps of three examples from Dataset 2 using the Grad-CAM algorithm. The left is the original image, the middle is the heatmap predicted by the dual-pretrained ResNet50 V2 model, and the right is a combination of the original image and predicted heatmap. (A) NAEH, (B) AEH, and (C) EC. Grad-CAM, Gradient-weighted Class Activation Mapping; NAEH, non-atypical endometrial hyperplasia; AEH, atypical endometrial hyperplasia; EC, endometrial cancer.

One NAEH image, one AEH image, and one EC image were randomly selected from Dataset 2 for visual interpretations using the Grad-CAM algorithm. The Grad-CAM-based heatmaps based on the convolutional layers of the dual pre-trained ResNet50 V2 model in Fig. 5A–C highlight the significant lesion regions. The closer the color of the lesion in the heatmap to red, the greater the weight, whereas the closer the color to blue, the smaller the weight.

### 3.4 Performance of the FSL models

Supplementary Materials Table S5 and Table S6 summarized the performance of the two FSL models in the QS. Compared with the FSL model combining the dual pretrained Xception, the FSL model combining the dual pretrained ResNet50 V2 eigenvectors extractor had a higher accuracy (0.878vs0.867), a higher macro precision (0.882vs0.867), and a higher macro F1-score (0.878vs0.867). When identifying AEH, the FSL model combining the dual pretrained ResNet50 V2 achieved a precision of 0.857, recall of 0.800, and F1-score of 0.828. When recognizing EC, the model achieved a precision of 0.964, recall of 0.900, and F1-score of 0.931, all outperforming the FSL model combining the dual pretrained Xception.

### 3.5 Comparison of the ensemble model, the traditional ResNet50 V2 model, the FSL model combining the dual pretrained ResNet50 V2 eigenvectors extractor, the junior sonographer, and the senior sonographer

**Fig. 6:**
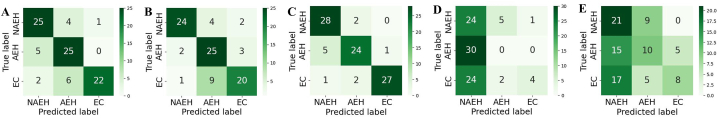
Confusion matrices of the Ensemble model (A), traditional ResNet50 V2 model (B), FSL model based on the dual pretrained ResNet50 V2 architecture (C), junior sonographer (D), and senior sonographer (E).

**Fig. 7:**
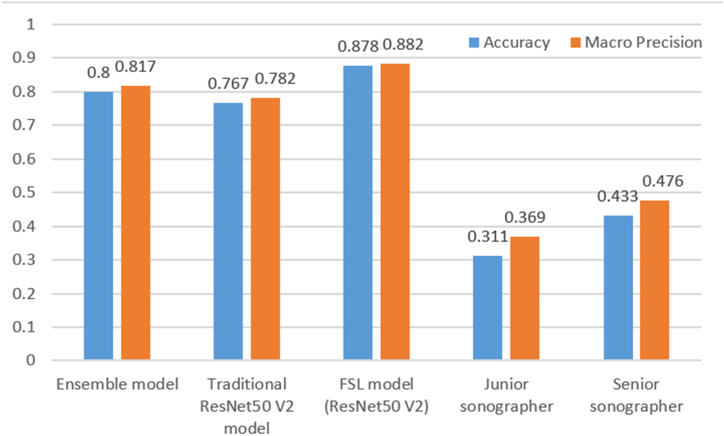
Bar chart of the ternary classification overall accuracy and macro precision of the Ensemble model, traditional ResNet50 V2 model, FSL model combining the dual pretrained ResNet50 V2 eigenvectors extractor, junior sonographer, and senior sonographer in the final test set.

Fig. 6A–E depicts the confusion matrices of the Ensemble model, traditional ResNet50 V2 model, 3-way 3-shot FSL model combining the dual pretrained ResNet50 V2 eigenvectors extractor, junior sonographer, and senior sonographer in the final test set. Among the three computer-aided tools, the FSL model correctly identified 28 NAEH, 24 AEH, and 27EC, with an accuracy of 0.878 and a macro precision of 0.882, outperforming the other two. Fig. 6D and E shows that the junior sonographer correctly identified 24 NAEH, 0 AEH, and 4 EC, and the senior sonographer correctly identified 21 NAEH, 10 AEH, and 8 EC, obviously inferior to the computer-aided tools. Taken together, the FSL was the best-performing model (Fig. 7).

The classification performances of the three computer-aided tools and two sonographers in recognizing each category (NAEH, AEH, EC) in the final test set are summarized in Table 2. When identifying NAEH, the traditional ResNet50 V2 model was the most precise (precision 0.889) with the FSL model exhibiting the best recall (0.933) and F1-score (0.875). When identifying AEH, the FSL model demonstrated the best precision (0.857) and F1-score (F1-score 0.828), with the best recall observed for the Ensemble and the traditional ResNet50 V2 models (recall 0.833). The FSL model achieved the best precision (0.964), the best recall (0.900), and the best F1-score (0.931) when identifying EC.

**Table 2:** The performance of the Ensemble model, traditional ResNet50 V2 model, FSL model (ResNet50 V2), junior sonographer, and senior sonographer in final test set.

		Group	Precision	Recall	F1-score
AutoML	Ensemble	NAEH	0.781	0.833	0.806
		AEH	0.714	0.833	0.769
		EC	0.957	0.733	0.830
Traditional DL	ResNet50 V2	NAEH	0.889	0.800	0.842
		AEH	0.658	0.833	0.735
		EC	0.800	0.667	0.727
FSL	ResNet50 V2	NAEH	0.824	0.933	0.875
		AEH	0.857	0.800	0.828
		EC	0.964	0.900	0.931
sonographer	Junior	NAEH	0.308	0.800	0.444
		AEH	0.000	0.000	0.000
		EC	0.800	0.133	0.229
sonographer	Senior	NAEH	0.396	0.700	0.506
		AEH	0.417	0.333	0.370
		EC	0.615	0.267	0.372

AutoML, automated machine learning; DL, deep learning; FSL, few-shot learning.

## 4 Discussion

AutoML, traditional DL, and FSL models were developed to identify NAEH, AEH, and EC based on the limited TVU images. The 3-way 3-shot FSL framework combining the dual pretrained ResNet50 V2 imaging eigenvectors extractor and KNN classifier demonstrates the best discrimination ability in the ternary classification task, outperforming the other two computer-aided diagnosis tools and two sonographers.

EC, the sixth most commonly occurring female cancer, is increasing in incidence and disease-associated mortality worldwide [29]. Early-stage (stage I or II) EC has a higher 5-year overall survival of 89.3 % in Asian women than the late-stage (III or IV) EC which has a 5-year overall survival of 41.2 % in Asian women [30]. The early diagnosis of EC was crucial. Although TVU is the preferred diagnostic method, it is difficult for physicians to distinguish accurately between AEH and EC. For example, the junior sonographer in this study only acquired a recall of 0 and 0.133 respectively in QS when identifying AEH and EC.

Machine learning (ML), as an objective and effective tool, has become a hot topic in disease diagnosis and evaluation. Zhang et al. [31] developed a radiomics model based on multimodal MRI to preoperatively distinguish concurrent endometrial carcinoma from atypical endometrial hyperplasia and achieved an AUC of 0.942. Huang et al. [32]established a combined nomogram by integrating clinical variables and radiomics features extracted from ultrasound images to predict 3-year disease-free survival of EC and acquired a favorable performance. By extracting a lot of the texture information of the tumor, radiomics features have been demonstrated reflecting the heterogeneity of tumor cells and tumor microenvironments [33,34]. Thus, we used MatLab software to extract 33 radiomics features to build six AutoML models, of which, the Ensemble model exhibited the best performance with accuracy and macro-precision of 0.758 and 0.771 respectively in Dataset 2. Furthermore, the LIME graphs show important the radiomics features in differentiating NAEH, AEH, and EC.

DL, as a further development of ML, is characterized by automatically learning from a large amount of image data and extracting discriminative features via convolutional operations. Unlike conventional radiomics which relies on the operator's expertise, DL can simplify steps by directly inputting a large amount of labeled images with greater reproducibility [35]. Furthermore, it can provide more in-depth information hiding in images via multiple processing layers [36]. Zhang et al. applied the tuned VGGNet-16 model to classify endometrial lesions based on hysteroscopic images and achieved an overall accuracy of 0.808. Fang et al. [37] proposed a self-supervised classification model for endometrial diseases based on ultrasound images, demonstrating enhanced performance compared to the baseline models. In our research, we applied dual pretrained ResNet50 V2 model to discriminate three categories of endometrial diseases (NAEH, AEH, and EC) and achieved an overall accuracy of 0.767 in the final test set, presenting a favorable performance. However, it is challenging to obtain a large amount of annotated training examples when applying DL for disease classification.

Few-shot learning is a novel algorithm in machine learning where a model is trained to learn from a very small number of samples. This is particularly useful in situations where obtaining a large amount of training data is difficult or expensive, such as in medical imaging or rare diseases. This approach is inspired by human learning, where we can learn new concepts quickly from just a few examples. Few-shot learning algorithms often employ transfer learning and metric learning. In the former approach, knowledge from a related task is transferred to the new task, or meta-learning, where the model learns to learn, improving its ability to adapt to new tasks with limited data. On the other hand, metric learning generates vector embeddings to represent given data samples, and make inferences by learning a function that measures distance metric representing the similarity between known samples and unknown samples.

FSL methods can maximize the information between feature representations and their corresponding labels using gained transferable knowledge from base classes. Currently, existing methods in FSL mainly focus on training meta-models based on the base data [38,39]. These trained meta-models are then applied to handle new FSL tasks involving disjoint target classes that have a limited dataset. However, recent studies demonstrated that approaches that train an effective feature extractor based on a pretrained classification network performed competitively in FSL tasks [[40], [41], [42]]. Yin et al. [42] proposed a 3-way 3-shot FSL framework combined with the dual pretrained EfficientNetV2-S feature extractor for the identification of rare gastric signet ring cell carcinoma (SRCC). The FSL model achieved the highest accuracy of 0.794 when identifying SRCC, showing a favorable performance. Suganya et al. [43] made use of the FSL technique combined with the ResNet-50 feature extractor to classify the severity of COVID-19 patients, achieving an average accuracy of 0.954. They adopted cosine similarity as a metric tool. In our research, we adopted a 3-way 3-shot FSL framework combined with dual pretrained ResNet50 V2 feature extractor. Also, we created a metric space based on the Euclidean distance and applied the KNN classifier to identify NAEH, AEH, and EC achieving a superior overall accuracy and macro precision to the automated machine learning models, traditional ResNet50 V2 models, junior sonographer, and senior sonographer.

Nonetheless, this study has several limitations. First, our FSL methods did not try different K sizes and lacked repeated samplings to avoid sampling error. Second, the handcrafted ROI inevitably deviate from the actual lesion morphology. Third, radiomics features were extracted from 2D rather than 3D TVU images, thus some important feature information may be lost. Fourth, we did not include more categories of endometrial diseases to validate the classification performance of FSL.

## 5 Conclusion

The 3-way 3-shot FSL framework combining the dual pretrained ResNet50 V2 eigenvectors extractor and KNN classifier performed well in distinguishing NAEH, AEH, and EC. The FSL method shows potential in computer-aided diagnosis for diseases with a limited dataset or low incidence.

## Ethics statement

This study was approved by the Ethics Committee of the First Affiliated Hospital of Soochow University on December 29, 2023 (Number: 2023-572).

## Informed consent statement

Since this study did not involve direct patient contact, only used clinical baseline data and medical images, and all medical data was anonymized, it was exempt from written informed consent and all participants were only required to provide verbal informed consent.

## Funding

This study was funded by the 10.13039/501100002949Jiangsu Province 10.13039/100000084Engineering Research Center of Precision Diagnostics and Therapeutics Development (No.SDGC2242) and the 10.13039/501100002949Jiangsu Province Sci-Tech Plan Special Fundation (No.BE2022729).

## Data availability statement

Data will be made available on request via corresponding author e-mail.

## CRediT authorship contribution statement

**Mingyue Wang:** Writing – original draft, Formal analysis, Data curation, Conceptualization. **Wen Liu:** Data curation. **Xinxian Gu:** Conceptualization. **Feng Cui:** Data curation. **Jin Ding:** Data curation. **Yindi Zhu:** Data curation. **Jinyan Bian:** Methodology, Formal analysis. **Wen Liu:** Writing – original draft, Methodology, Formal analysis, Conceptualization. **Youguo Chen:** Writing – review & editing, Funding acquisition, Conceptualization. **Jinhua Zhou:** Writing – review & editing, Project administration, Funding acquisition.

## Declaration of competing interest

The authors declare that they have no competing interests.

## **Abbreviations**:

**NAEH**: non-atypical endometrial hyperplasia
**AEH**: atypical endometrial hyperplasia
**EC**: endometrial cancer
**TVU**: transvaginal ultrasonic
**FSL**: few-shot learning
**SS**: support set
**QS**: query set
**KNN**: k-nearest neighbor
**DL**: Deep learning
**AutoML**: automated machine learning
**ROI**: region of interest
**LIME**: Local interpretable model-agnostic explanations
**Grad-CAM**: Gradient-weighted Class Activation Mapping
**ML**: Machine learning
