# Supplementary material for: Few-shot learning to identify atypical endometrial hyperplasia and endometrial cancer based on transvaginal ultrasonic images
Source: Heliyon. 2024 Aug 16;10(16):e36426. doi: 10.1016/j.heliyon.2024.e36426 (PMC11381780; doi:10.1016/j.heliyon.2024.e36426)
Supplement: Multimedia component 1 [file mmc1.docx]

| **Table S1.** The overall performance of six AutoML models in the ternary classification task in Dataset 2. | | | |
| --- | --- | --- | --- |
| AutoML | Accuracy | Macro Precision | Macro F1-score |
| Ensemble | 0.758 | 0.771 | 0.759 |
| DL | 0.737 | 0.745 | 0.737 |
| XGBoost | 0.747 | 0.750 | 0.747 |
| GLM | 0.677 | 0.678 | 0.677 |
| GBM | 0.646 | 0.647 | 0.647 |
| RF | 0.596 | 0.596 | 0.594 |

| **Table S2.** The performance of the AutoML models in identifying each of the three categories (NAEH, AEH, EC) in Dataset 2. | | | | |
| --- | --- | --- | --- | --- |
| AutoML | Group | Precision | Recall | F1-score |
| Ensemble | NAEH | 0.743 | 0.788 | 0.765 |
|  | AEH | 0.684 | 0.788 | 0.732 |
|  | EC | 0.885 | 0.697 | 0.780 |
| DL | NAEH | 0.735 | 0.758 | 0.746 |
|  | AEH | 0.684 | 0.788 | 0.732 |
|  | EC | 0.815 | 0.667 | 0.733 |
| XGBoost | NAEH | 0.722 | 0.788 | 0.754 |
|  | AEH | 0.735 | 0.758 | 0.746 |
|  | EC | 0.793 | 0.697 | 0.742 |
| GLM | NAEH | 0.688 | 0.667 | 0.677 |
|  | AEH | 0.629 | 0.667 | 0.647 |
|  | EC | 0.719 | 0.697 | 0.708 |
| GBM | NAEH | 0.636 | 0.636 | 0.636 |
|  | AEH | 0.618 | 0.636 | 0.627 |
|  | EC | 0.688 | 0.667 | 0.677 |
| RF | NAEH | 0.605 | 0.697 | 0.648 |
|  | AEH | 0.516 | 0.485 | 0.500 |
|  | EC | 0.667 | 0.606 | 0.635 |

| **Table S3.** The overall performance of two traditional DL models in the ternary classification task in Dataset 2. | | | |
| --- | --- | --- | --- |
| Traditional DL models | Accuracy | Macro Precision | Macro F1-score |
| ResNet50 V2 | 0.737 | 0.757 | 0.738 |
| Xception | 0.737 | 0.741 | 0.737 |

| **Table S4.** The performance of two traditional DL models in identifying each of the three categories (NAEH, AEH, EC) in Dataset 2. | | | | |
| --- | --- | --- | --- | --- |
| Traditional DL models | Group | Precision | Recall | F1-score |
| ResNet50 V2 | NAEH | 0.857 | 0.727 | 0.787 |
|  | AEH | 0.636 | 0.848 | 0.727 |
|  | EC | 0.778 | 0.636 | 0.700 |
| Xception | NAEH | 0.781 | 0.758 | 0.769 |
|  | AEH | 0.684 | 0.788 | 0.732 |
|  | EC | 0.759 | 0.667 | 0.710 |

| **Table S5.** The overall performance of two FSL models in the ternary classification task in the query set. | | | |
| --- | --- | --- | --- |
| FSL models | Accuracy | Macro Precision | Macro F1-score |
| ResNet50 V2 | 0.878 | 0.882 | 0.878 |
| Xception | 0.867 | 0.867 | 0.867 |

| **Table S6.** The performance of two FSL models in identifying each of the three categories (NAEH, AEH, EC) in the query set. | | | | |
| --- | --- | --- | --- | --- |
| FSL models | Group | Precision | Recall | F1-score |
| ResNet50 V2 | NAEH | 0.824 | 0.933 | 0.875 |
|  | AEH | 0.857 | 0.800 | 0.828 |
|  | EC | 0.964 | 0.900 | 0.931 |
| Xception | NAEH | 0.844 | 0.900 | 0.871 |
|  | AEH | 0.862 | 0.833 | 0.847 |
|  | EC | 0.897 | 0.867 | 0.881 |


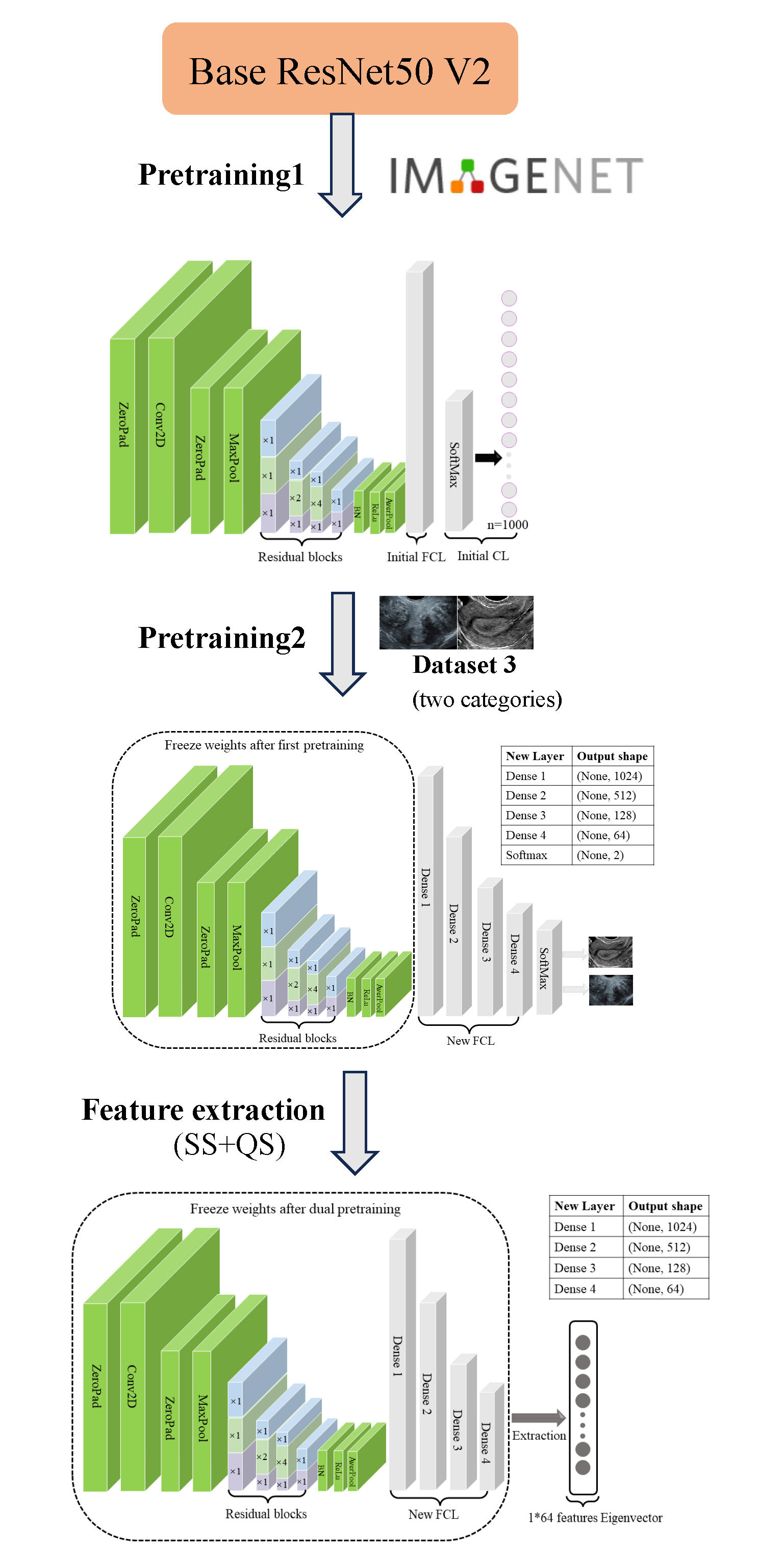


**Figure S1.** The process of dual pretraining in base ResNet50 V2 architecture and feature extraction in support set (SS) and query set (QS).


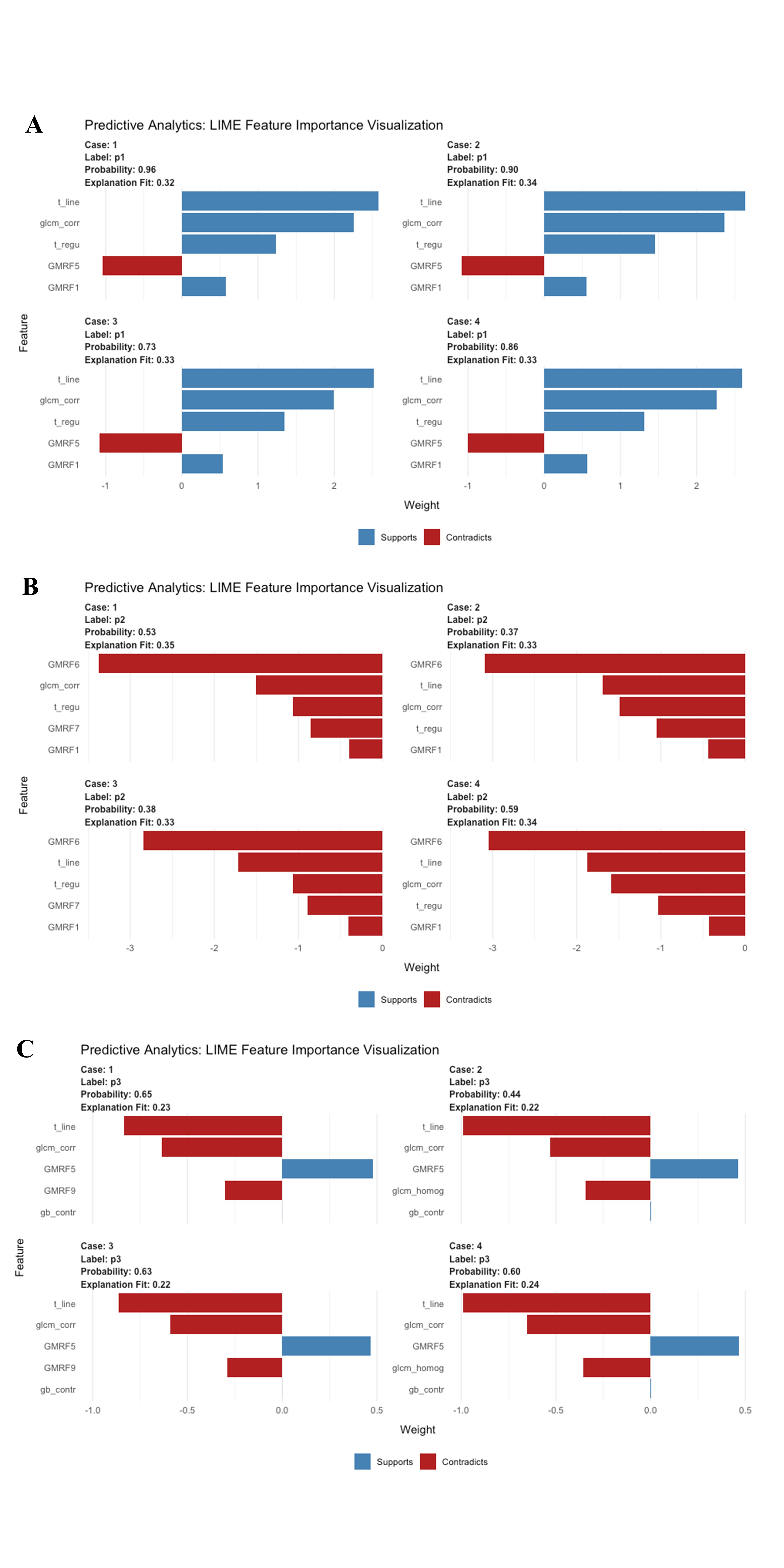


**Figure S2.** The LIME graphs of the Ensemble model in sixteen cases from Dataset 2. (A) four NAEH cases. (B) four AEH cases. (C) four EC cases. The LIME graphs show how the important radiomics features contributed to the diagnosis predicted by the Ensemble model. Label p1 means NAEH. Label p2 means AEH. Label p3 means EC. LIME, local interpretable model agnostic explanation; NAEH, non-atypical endometrial hyperplasia; AEH, atypical endometrial hyperplasia; EC, endometrial cancer.
